# Supplementary material for: Trace gas oxidation sustains energy needs of a thermophilic archaeon at suboptimal temperatures
Source: Nat Commun. 2024 Apr 15;15:3219. doi: 10.1038/s41467-024-47324-2 (PMC11018855; doi:10.1038/s41467-024-47324-2)
Supplement: Supplementary file 1 — Supplementary information [file 41467_2024_47324_MOESM1_ESM.pdf]

Supplementary Information

Trace gas oxidation sustains energy needs of a thermophilic archaeon at suboptimal temperatures

Pok Man Leung[1 *], Rhys Grinter[1], Eve Tudor-Matthew[1], James P. Lingford[1], Luis Jimenez[1], Han-Chung Lee[2], Michael Milton[1], Iresha Hanchapola[2], Erwin Tanuwidjaya[2], Ashleigh Kropp[1], Hanna A. Peach[3], Carlo R. Carere[3,4], Matthew B. Stott[3,5], Ralf B. Schittenhelm[2], Chris Greening[1 *]

[1] Department of Microbiology, Biomedicine Discovery Institute, Monash University, Clayton, VIC 3800, Australia

[2] Monash Proteomics and Metabolomics Platform and Department of Biochemistry, Monash Biomedicine Discovery Institute, Monash University, Clayton, VIC 3800, Australia

[3] Geomicrobiology Research Group, Department of Geothermal Sciences, Te Pū Ao | GNS Science, Wairakei, Taupō 3377, Aotearoa-New Zealand

[4] Te Tari Pūhanga Tukanga Matū | Department of Chemical and Process Engineering, Te Whare Wānanga o Waitaha | University of Canterbury, Christchurch 8140, Aotearoa-New Zealand

[5] Te Kura Pūtaiao Koiora | School of Biological Sciences, Te Whare Wānanga o Waitaha | University of Canterbury, Christchurch 8140, Aotearoa-New Zealand

[*] **Corresponding authors:** Pok Man Leung (bob.leung@monash.edu), Chris Greening (chris.greening@monash.edu)

Supplemental Note 1 – AlphaFold modelling of the *Acidianus brierleyi* hydrogenases

Hca - Group 1g hydrogenase

To generate the structural model of Hca from *A. brierleyi,* the amino acid sequences of the four putative structural subunits HcaS, HcaL, Isp1-1, and Isp2-1 were provided to AlphaFold multimer. AlphaFold convincingly assembled all four proteins into a single complex, supporting previous work indicating that HcaS, HcaL and Isp1-1 form a complex in *Acidianus ambivalens* [1]. As predicted based on sequence homology, Isp1-1 is an integral membrane di-heme *b*-type cytochrome protein that forms a complex with the extracellular [NiFe]-hydrogenase catalytic subunit formed by HcaS and HcaL, which is anchored to the membrane by a transmembrane helix at the C-terminus of HcaS. Based on homology to the NarI subunit of nitrate reductase, we modelled two heme molecules in the integral membrane region of Isp1, both exhibiting bidentate coordination by conserved histidine residues (**Fig. 4B**). Interaction between Isp1 and HcaS positions one of these hemes within electron transfer distance of the distal [4Fe-4S]-cluster of HcaS, providing a route for electrons produced by H_2 oxidation by HcaL. In hydrogenases and related enzymes, *b*-type cytochromes generally reduce or oxidize respiratory quinone, providing a link between the enzyme and the cellular electron transport chain [2–4] (**Fig. 4B**). As our proteomic data shows Hca primarily functions as a H_2-oxidizing enzyme during anaerobic sulfur-reducing metabolism, it is likely that Isp1 reduces quinone. Intriguingly, the previously uncharacterized Isp2-1 subunit forms a complex with Isp1-1 on the cytoplasmic side of the membrane. Isp2-1 contains three iron-sulfur clusters, two of which have a cubane [4Fe-4S] configuration, and one of which resembles the non-cubane [4Fe-4S] from the HdrBC subunits of CoB-CoM heterodisulfide reductase, which Isp2-1 is structurally homologous to [5]. The role of Isp2 in Hca function requires experimental verification. However, this model indicates that it provides a conduit for electrons derived from H_2 oxidation to the cytoplasm, where they could be used to reduce NAD^+ or ferredoxin to drive carbon fixation and other biosynthetic processes during autotrophic growth. The dual path for electrons within Hca creates the possibilities that electron path alternation in response to H_2 concentrations and redox conditions or electron bifurcation may occur, with electrons from H_2 split between relatively high potential quinone and a low potential electron acceptor like ferredoxin or NAD^+. A potential mechanism for the latter to occur in such hydrogenases may be through quinone-based electron bifurcation where quinol reduced by H_2 acts as the bifurcation intermediate for both ferredoxin and quinone. The dual-heme arrangement of Hca is also reminiscent of the complex III known to mediate Q-cycle.

Hsu2 - SUL2 hydrogenase

While Hsu2 is from a distinct phylogenetic lineage to Hca (**Fig. 3**), based on its associated genes, its subunit composition appears to be analogous to Hca, consisting of HsuS2, HsuL2, Isp1-2, and Isp2-2 subunits (**Fig. 4A**). Consistent with this, the model of Hsu2 produced by AlphaFold is similar to Hca with an extracellular membrane-anchored [NiFe]-hydrogenase module composed of HsuS2 and HsuL2 interacting with the integral membrane Isp1-2, with heme and FeS cluster distances compatible with electron transfer. As with Hca, the Isp2-2 forms a complex with Isp1-2 on the cytoplasmic side of the membrane. Isp2-2 only coordinates two [4Fe-4S] clusters, lacking the predicted cubane cluster present in Isp2-1 (**Fig. 4C**). Despite this, like Isp2-1 in Hca, Isp2-2 provides a conduit for electrons from H_2 oxidation by Hsu to the cytoplasm, possibly for the direct reduction of substrates utilized for carbon fixation. The similarity of the structural models of Hca and Hsu2 is intuitive given their distinct expression profiles and likely allows these two proteins to fulfil analogous roles, with Hca acting under anaerobic conditions with sulfur as the electron acceptor and Hsu2 acting under aerobic conditions with O_2 as the electron acceptor.

Hys – Group 2e hydrogenase

Upstream of the genes encoding the Hys [NiFe]-hydrogenase catalytic subunits HysS and HysL, and encoded in the opposite direction, is a 165 amino acid protein distantly related to the HucM subunit of Huc, a group 2a hydrogenase produced by *Mycobacterium smegmatis* (**Fig. 4A**). Recent structural analysis of Huc shows that HucM forms a tetramer that acts as a scaffold, forming a complex with four dimers of the HucSL [NiFe]-hydrogenase catalytic subunit. Each catalytic subunit is composed of HucS and HucL proteins, with the Huc complex containing four HucM, eight Huc and eight HucS subunits [6]. Based on this arrangement, we hypothesized that the Hys catalytic subunits form a dimer, which was confirmed by the AlphaFold model produced when the program was provided with two HysS and two HysL subunits. This structure is highly similar to the catalytic dimer of HucSL (RMSD = 1.55 Å for 5613/8158 atoms) and contains conserved amino acids responsible for coordinating menaquinone at the Huc electron acceptor site (**Fig. 4D**). Interestingly, when AlphaFold was provided with four HysM subunits, it produced a tetrameric coiled-coil tube that is topologically and structurally similar to HucM (**Fig. 4E**). Moreover, like HucM, the HysM tube is exclusively lined with hydrophobic residues. As well as serving as a scaffold for the Huc complex, the end of the HucM tube interacts with the cytoplasmic membrane, acting as a conduit allowing hydrophobic menaquinone to reach the electron acceptor site in HucS [6]. The analogous structure and hydrophobic nature of the HysM tube suggest that it plays a similar role in Hys,

which is remarkable considering the distant relationship between archaeal Hys and bacterial Huc. Unlike HucM, the AlphaFold model of HysM forms a compact tube, which lacks an obvious region that would facilitate the binding of Hys catalytic dimers. However, this may be a limitation of the AlphaFold modelling, as due to memory constraints we were not able to model all subunits concurrently. Alternatively, an additional unidentified subunit may be required to scaffold the complex. However, no candidates were identified by AlphaFold modelling of protein encoded by genes in proximity to HysS or HysL. Based on the similarity of Hys to Huc, we have generated a plausible model of the complete Hys complex (**Fig. 4E**).

Hsu1 – SUL1 hydrogenase

The Hsu1 gene cluster contains genes encoding the [NiFe]-hydrogenase catalytic HsuS1 and HsuL1 subunits (**Fig. 4A**). HusS1 is truncated compared to other [NiFe]-hydrogenase small subunits and, as a result, only contains a single [4Fe-4S]-cluster (**Fig. S4A**). Based on these subunits, it is unclear what the electron carrier for Hsu1 is. However, upstream of the HsuS1 gene are the three open reading frames encoding the proteins SdhB2, SdhC2, and SdhD2 (a gene encoding SdhA-like protein was not identified next to the three genes), homologous to the three subunits of succinate dehydrogenase. In succinate dehydrogenase, these subunits form a [FeS]-cluster and heme-containing integral membrane complex that relays electrons between the succinate oxidizing subunit and membrane-bound quinone [7]. It is possible that these proteins serve as the electron relay from the Hsu1 catalytic subunit, meaning that Hsu1 also reduces quinone. However, while AlphaFold modelling placed the subunits within plausible electron transfer distance, a stable interface between the HsuS1/HsuL1 and SdhB2/SdhC2/ SdhD2 was not predicted (**Fig. S4B**). This makes it unclear if these proteins are functionally related.

Supplementary Tables

Table S1. Structures used to model cofactors of hydrogenases from *Acidianus brierleyi*.

Hydrogenase	Subunits	Reference (PDB entries)	Reference description
Group 1g	HcaS	5AA5 chain A	5AA5 - Actinobacterial-type [NiFe]-hydrogenase from *Ralstonia eutropha*
	HcaL	5AA5 chain C	
	Isp1_1	3EGW chain C	3EGW - The crystal structure of the NarGHI mutant NarH
	Isp2_1	7BKB chains P/Q	7BKB – Formate dehydrogenase-heterodisulfide reductase - formylmethanofuran dehydrogenase complex from *Methanospirillum hungatei*
Group 2e	HysS	8DQV chain B	8DQV - CryoEM structure of the [NiFe]-hydrogenase Huc from *Mycobacterium smegmatis*
	HysL	8DQV chain A	
	HysM	N/A	N/A
SUL1	HsuS1	4C3O chain B	4C3O - Structure and function of an oxygen tolerant [NiFe]-hydrogenase from *Salmonella*
	HsyL1	4C3O chain B	
	SdhB2	1NEK chain B	1NEK - Complex II (Succinate dehydrogenase) from *E. coli* with ubiquinone bound
	SdhC2	1NEK chain C	
	SdhD2	1NEK chain D	
SUL2	HsuS2	4C3O chain B	4C3O - Structure and function of an oxygen tolerant [NiFe]-hydrogenase from *Salmonella*
	HsuL2	4C3O chain B	
	Isp1_2	3EGW chain C	3EGW - The crystal structure of the NarGHI mutant NarH
	Isp2_2	7BKB chains P/Q	7BKB – Formate dehydrogenase-heterodisulfide reductase - formylmethanofuran dehydrogenase complex from *Methanospirillum hungatei*

Supplementary Figures

Fig. S1. Aerobic oxidation of atmospheric hydrogen (H_2) in ambient air during exponential growth of *Acidianus brierleyi* at 70°C and 25°C. Gas chromatography measurement of consumption of ambient H_2 (average 0.62 ppmv in lab air) by *A. brierleyi* grown at mid-exponential growth at 70°C and 25°C, with heat-killed cells as negative controls. Enclosed shows the abiotic production of H_2 in heat-killed control vial at 70°C, likely due to thermal degradation of butyl-rubber stopper as previously observed [8]. Dotted line indicates the reported global average atmospheric H_2 mixing ratio (0.53 ppmv) [9]. Data are presented as mean ± S.D. values of three biological replicates (n = 3).

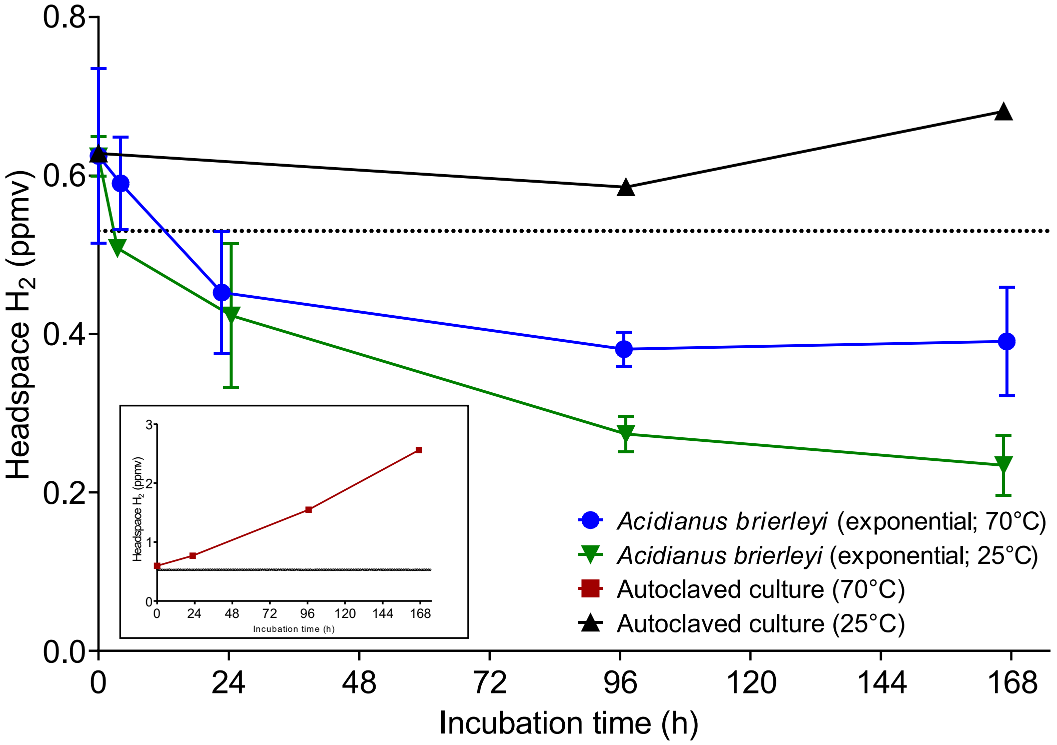

Fig. S2. Time course of internal standard methane (CH_4) concentrations in *Acidianus brierleyi* cultures (A) and negative controls (B) used in gas chromatography experiments. Headspace CH_4 mixing ratio is presented on a logarithmic scale.

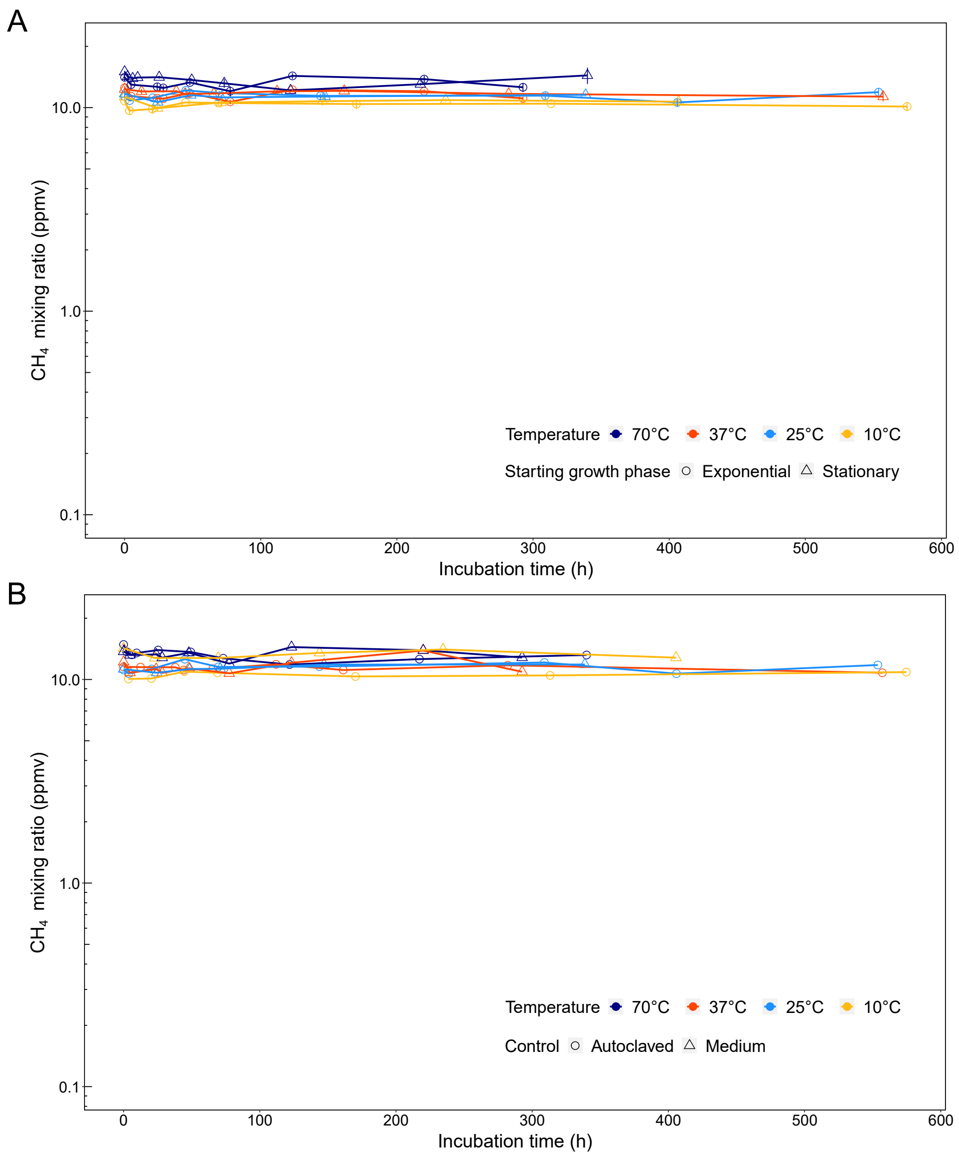

Fig. S3. Measurement of viability proxies of stationary phase cultures of *Acidianus brierleyi* maintained at low headspace H_2 levels at 70°C and 25°C. Measurement of (**A**) optical density at 600 nm (OD_{600}); (**B**) cell protein in culture (µg / ml); (**C**) ATP level in culture (nM); and (**D**) ATP level normalized by cell protein (nmol / $mg_{protein}$) in stationary phase cultures of *A. brierleyi* supplemented daily with low levels of headspace H_2 within twenty days at 70°C and 25°C. For all panels, data are presented as mean ± S.D. values of three biological replicates (n = 3) while asterisks indicate significantly higher levels in H_2-supplemented cultures than cultures with no extra H_2 at the same timepoint based on one-sided Student's t-test (* $p < 0.05$; ** $p < 0.005$).

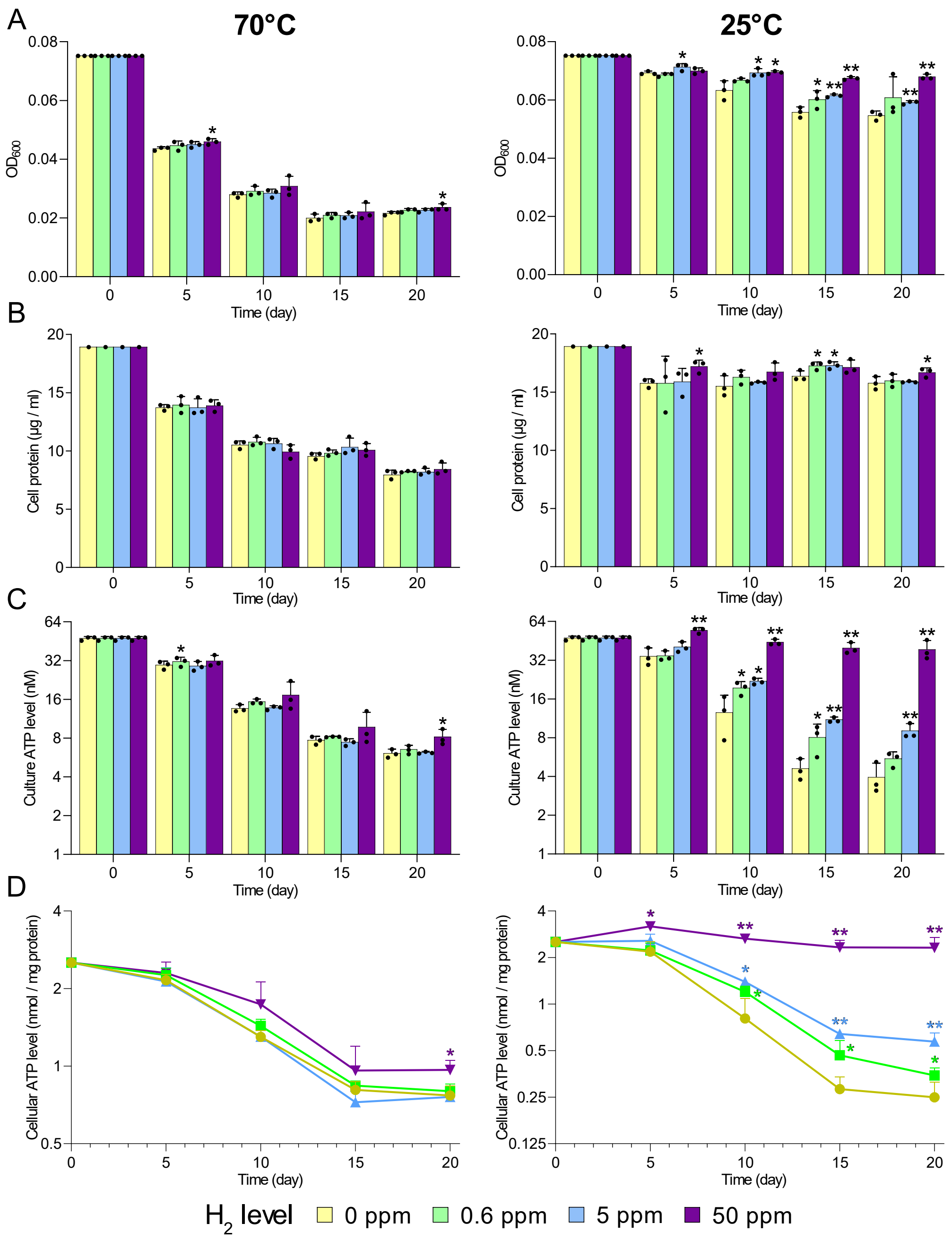

Fig. S4. AlphaFold modelling of Hsu1. (**A**) An AlphaFold model of the HsuS1 and HsuL1 subunits shown as a cartoon representation (**upper panel**) and the electron transport relay formed by modelled cofactors, with predicted electron donors and acceptors indicated (**bottom panel**). (**B**) An AlphaFold model of a complex between Hsu1 and the SdhB2, SdhC2 and SdhD2 proteins, which are encoded upstream of the HsuS1 subunit. An intimate complex was not predicted between HsuS1/HsuL1 and SdhB2/SdhC2/SdhD2. However, an [FeS]-cluster of both complexes is within electron transfer distance in the model suggesting SdhB2/SdhC2/SdhD2 may accept electrons from Hsu1 for the reduction of quinone via a transient interaction between the two complexes.

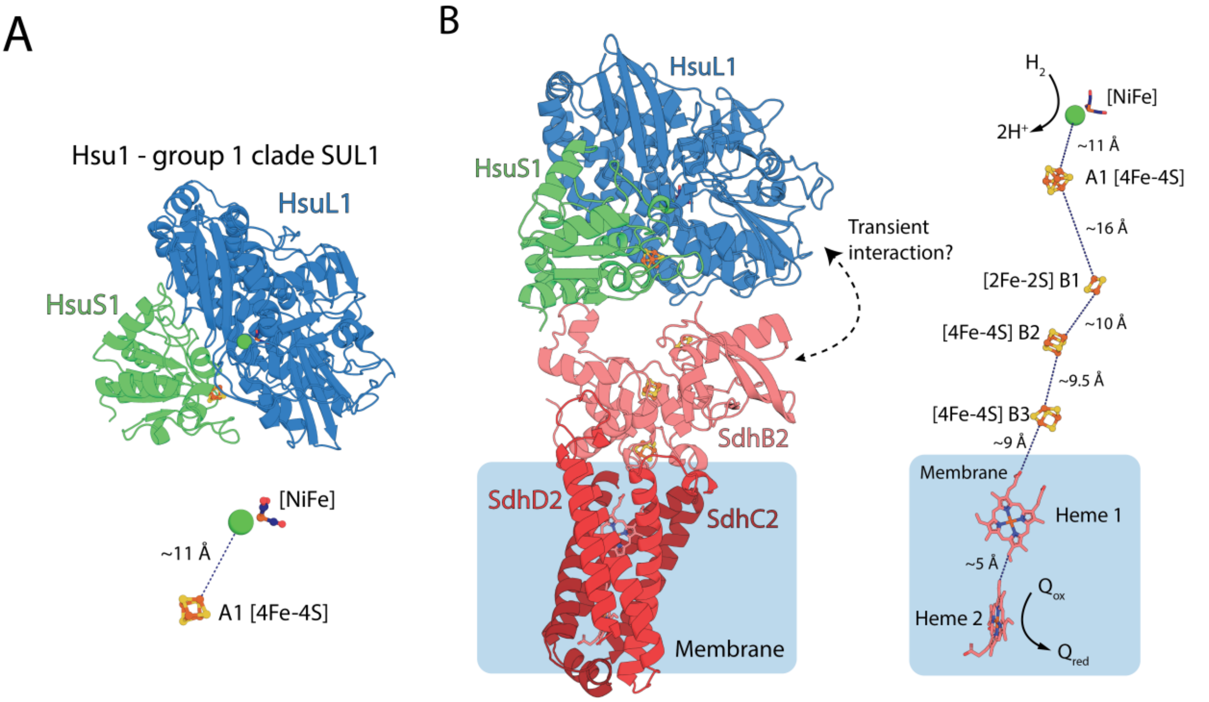

Fig. S5. Original blue native PAGE gel images as in Fig. 5A. *Acidianus brierleyi* cells were harvested during aerobic organotrophic exponential growth in a headspace with either 20% H_2 or ambient H_2. Whole cell lysates, cytosols, and membranes were separated by blue native polyacrylamide gel electrophoresis and stained with Coomassie blue (left panel) and artificial electron acceptor nitrotetrazolium blue chloride (NBT) under a 7%-H_2 atmosphere (right panels). 10, 5, and 2.5 µl of each fraction were loaded on the gel. The protein ladder in the left lane was stained with Coomassie blue. Purple bands indicated by arrows suggest reduction of NBT by hydrogenase activity. The absence of positive bands in whole cell lysate likely reflects the dilute protein concentration as suggested by Coomassie stain.

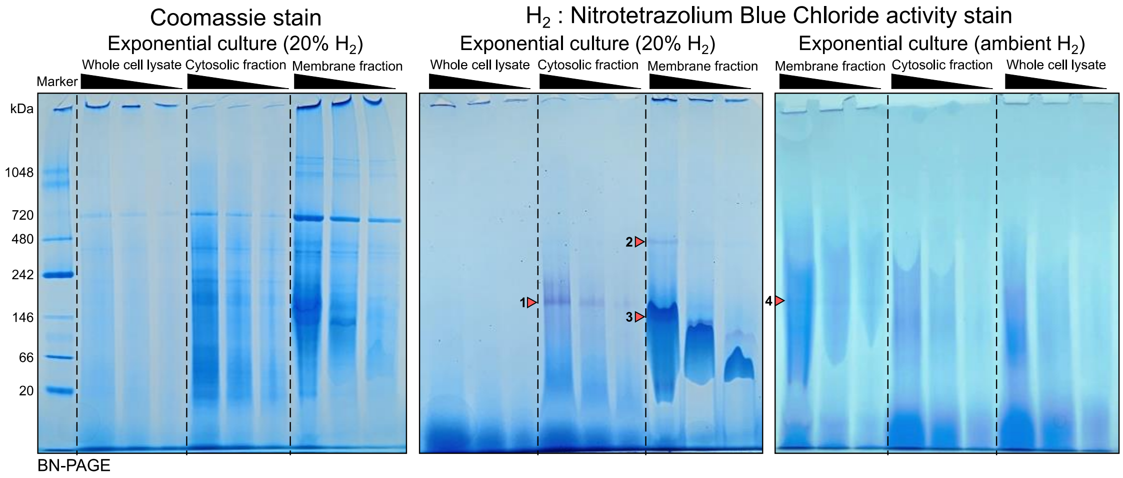

Fig. S6. Carbon monoxide dehydrogenase operon of *Acidianus brierleyi*.

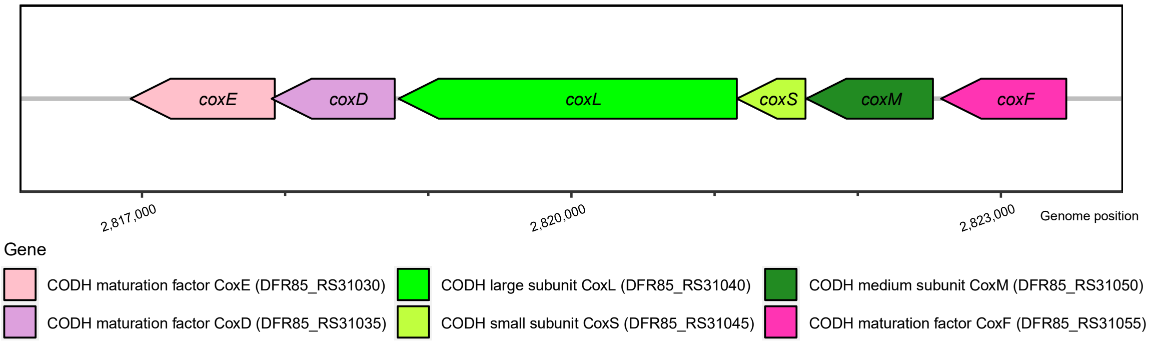

Fig. S7. Maximum-likelihood phylogenetic tree showing the evolutionary history of the catalytic subunit of the form I CO dehydrogenase (CoxL). Evolutionary distances were computed using the Poisson correction model, gaps were treated by partial deletion, and the tree was bootstrapped with 50 replicates. The tree was constructed using a representative subset of CoxL amino acid sequences from ref. [10]. The major clades are color-shaded and the sequences from Thermoproteota including *A. brierleyi* are labelled in red text.

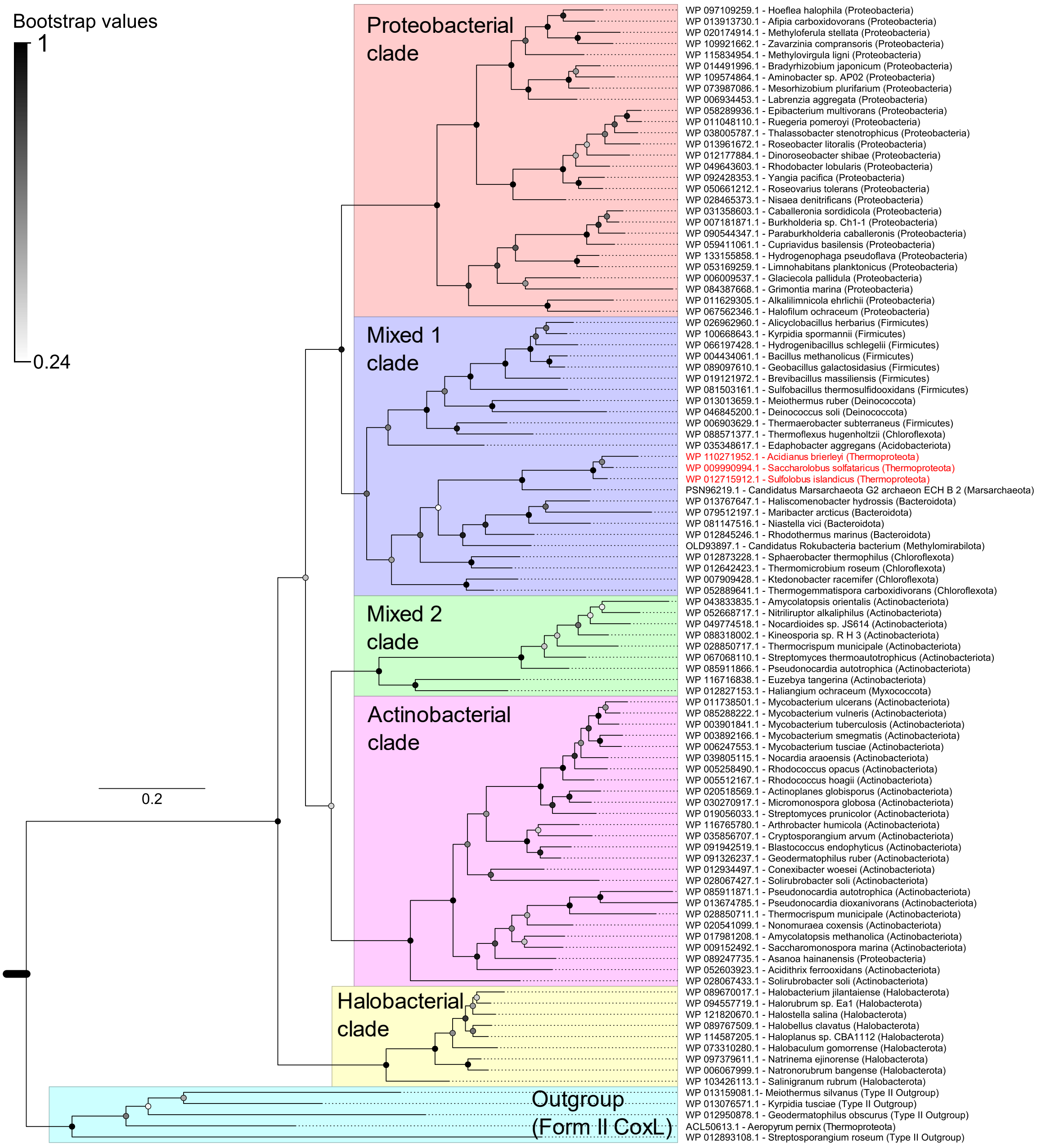

Fig. S8. Principal component analysis (PCA) showing distinct proteome clustering of *Acidianus brierleyi* grown at four different conditions. Mid-exponential growth on heterotrophic medium (EX); stationary phase on heterotrophic medium (ST); transition from heterotrophic to sulfur-dependent anaerobic hydrogenotrophic growth on mineral medium (AN); transition from heterotrophic to aerobic hydrogenotrophic growth on mineral medium (AE).

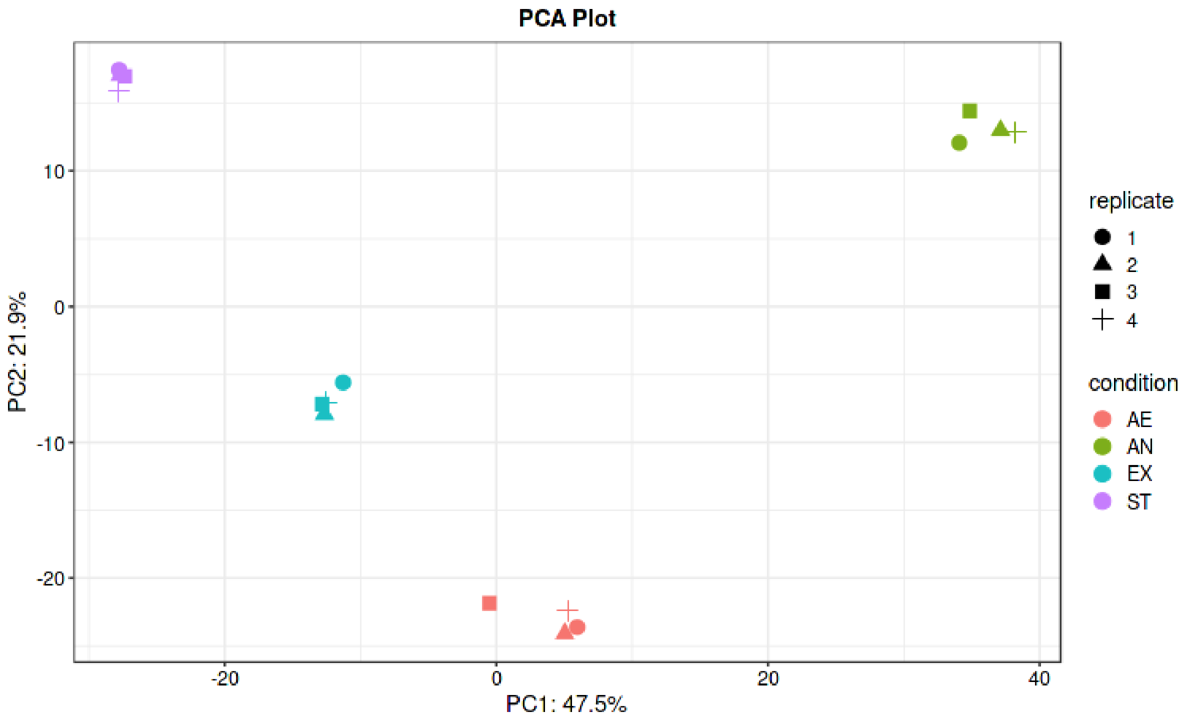

Fig. S9. Quantitative comparison of *Acidianus brierleyi* proteins involved in hydrogenase maturation, sulfur oxidation and iron uptake/oxidation under heterotrophic growth, stationary phase, sulfur-dependent hydrogenotrophic growth and aerobic hydrogenotrophic growth. Culture condition (four biological replicates each): EX, mid-exponential growth phase on heterotrophic medium; ST, stationary phase on heterotrophic medium; AN, anaerobic sulfur-dependent hydrogenotrophic growth; AE, aerobic hydrogenotrophic growth (**Methods**). Normalised protein abundance value represents MaxLFQ total intensity for the protein. Bubble size and color indicate protein abundance of the corresponding gene product in each biological replicate. Significant difference in fold changes of protein abundance of each condition pair is denoted by asterisks (two-sided Student's t-test with Benjamini-Hochberg correction, adjusted p value ⩽0.001, ***; ⩽0.01, **; ⩽0.05, *; > 0.05, ns).

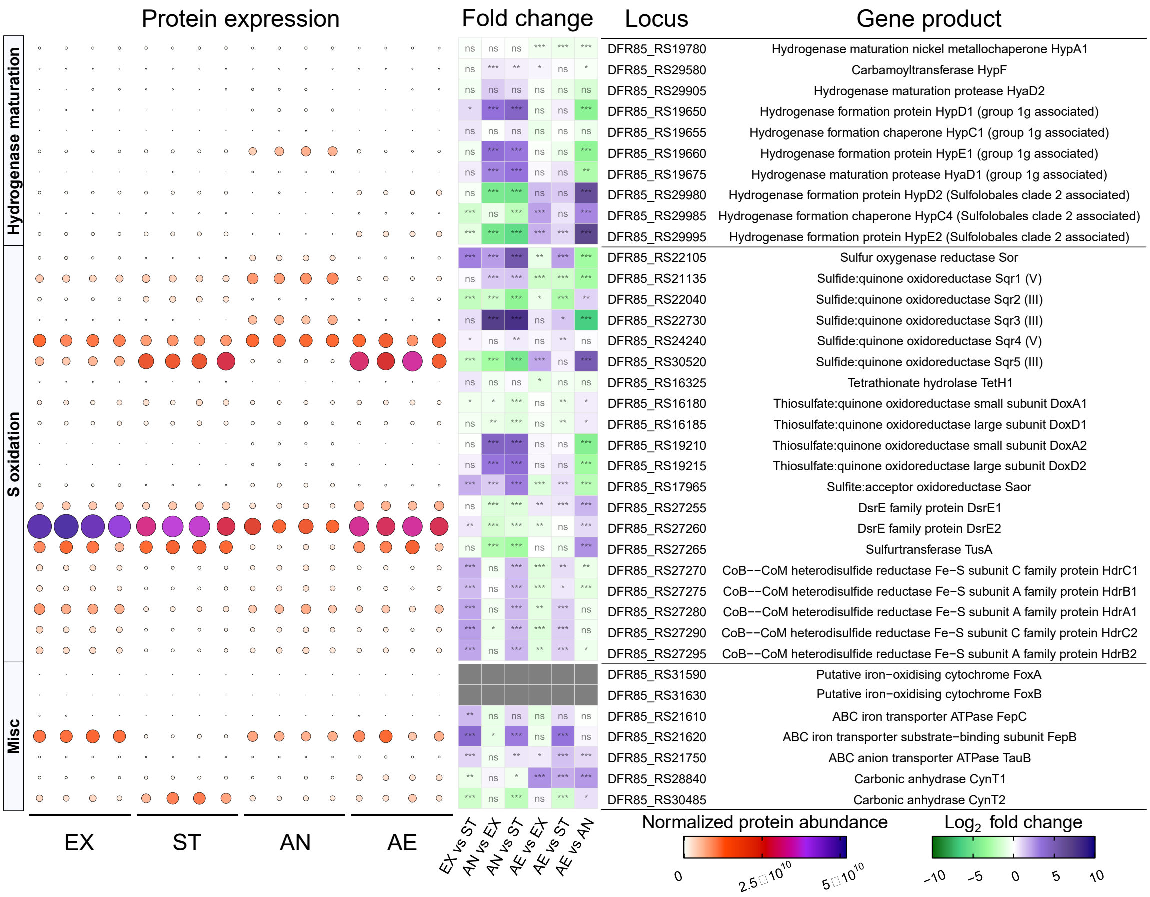

Fig. S10. Quantitative comparison of *Acidianus brierleyi* proteins involved in carbon reserve degradation and amino acid/peptide/sugar/acid transport under heterotrophic growth, stationary phase, sulfur-dependent hydrogenotrophic growth and aerobic hydrogenotrophic growth. Culture condition (four biological replicates each): EX, mid-exponential growth phase on heterotrophic medium; ST, stationary phase on heterotrophic medium; AN, anaerobic sulfur-dependent hydrogenotrophic growth; AE, aerobic hydrogenotrophic growth (**Methods**). Normalised protein abundance value represents MaxLFQ total intensity for the protein. Bubble size and color indicate protein abundance of the corresponding gene product in each biological replicate. Significant difference in fold changes of protein abundance of each condition pair is denoted by asterisks (two-sided Student's t-test with Benjamini-Hochberg correction, adjusted p value $\leqslant$0.001, ***; $\leqslant$0.01, **; $\leqslant$0.05, *; > 0.05, ns).

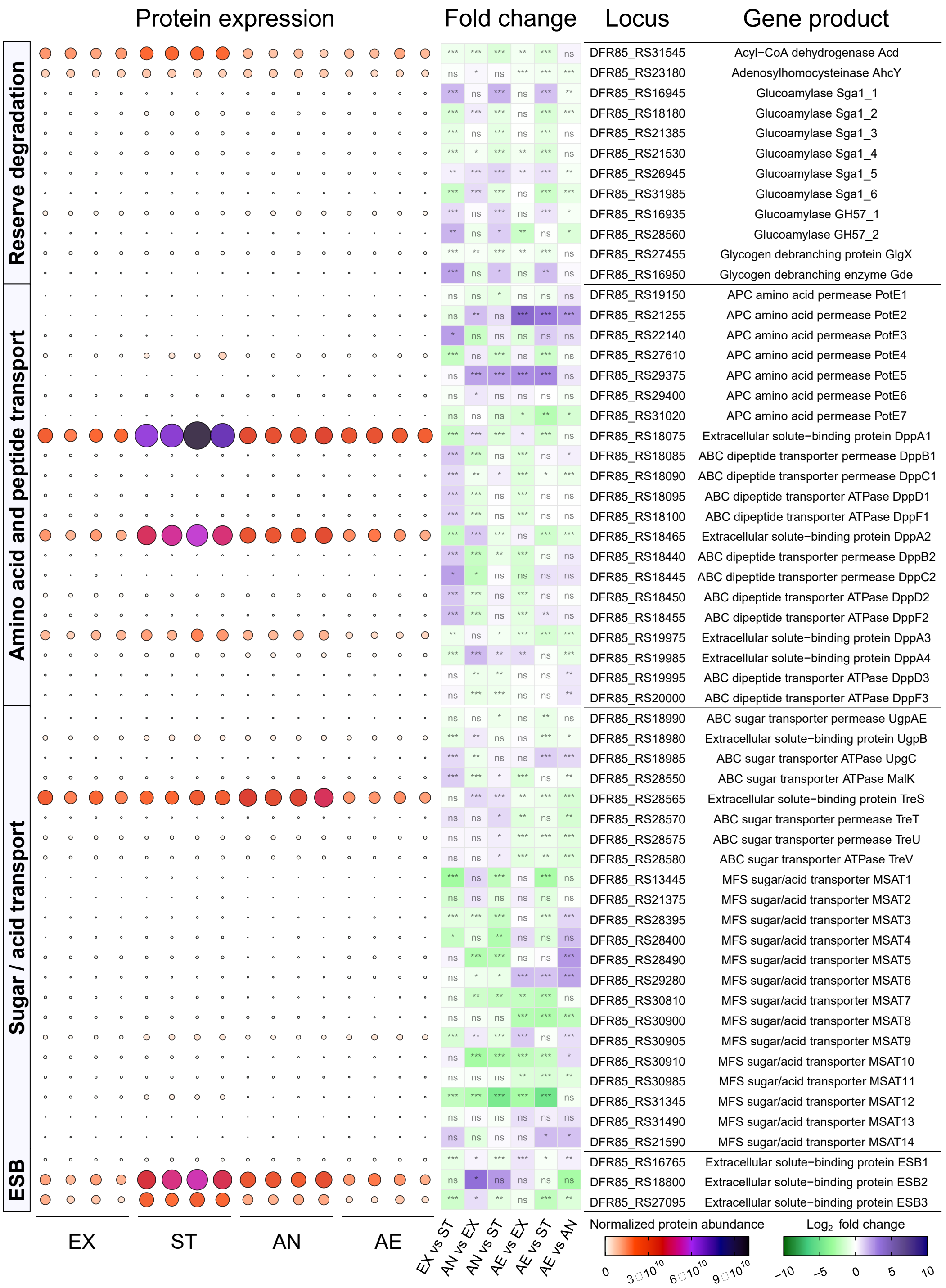

Protein expression
Fold change
Locus
Gene product
Reserve degradation
Amino acid and peptide transport
Sugar / acid transport
ESB
DFR85_RS31545 Acyl−CoA dehydrogenase Acd
DFR85_RS23180 Adenosylhomocysteinase AhcY
DFR85_RS16945 Glucoamylase Sga1_1
DFR85_RS18180 Glucoamylase Sga1_2
DFR85_RS21385 Glucoamylase Sga1_3
DFR85_RS21530 Glucoamylase Sga1_4
DFR85_RS26945 Glucoamylase Sga1_5
DFR85_RS31985 Glucoamylase Sga1_6
DFR85_RS16935 Glucoamylase GH57_1
DFR85_RS28560 Glucoamylase GH57_2
DFR85_RS27455 Glycogen debranching protein GlgX
DFR85_RS16950 Glycogen debranching enzyme Gde
DFR85_RS19150 APC amino acid permease PotE1
DFR85_RS21255 APC amino acid permease PotE2
DFR85_RS22140 APC amino acid permease PotE3
DFR85_RS27610 APC amino acid permease PotE4
DFR85_RS29375 APC amino acid permease PotE5
DFR85_RS29400 APC amino acid permease PotE6
DFR85_RS31020 APC amino acid permease PotE7
DFR85_RS18075 Extracellular solute−binding protein DppA1
DFR85_RS18085 ABC dipeptide transporter permease DppB1
DFR85_RS18090 ABC dipeptide transporter permease DppC1
DFR85_RS18095 ABC dipeptide transporter ATPase DppD1
DFR85_RS18100 ABC dipeptide transporter ATPase DppF1
DFR85_RS18465 Extracellular solute−binding protein DppA2
DFR85_RS18440 ABC dipeptide transporter permease DppB2
DFR85_RS18445 ABC dipeptide transporter permease DppC2
DFR85_RS18450 ABC dipeptide transporter ATPase DppD2
DFR85_RS18455 ABC dipeptide transporter ATPase DppF2
DFR85_RS19975 Extracellular solute−binding protein DppA3
DFR85_RS19985 Extracellular solute−binding protein DppA4
DFR85_RS19995 ABC dipeptide transporter ATPase DppD3
DFR85_RS20000 ABC dipeptide transporter ATPase DppF3
DFR85_RS18990 ABC sugar transporter permease UgpAE
DFR85_RS18980 Extracellular solute−binding protein UgpB
DFR85_RS18985 ABC sugar transporter ATPase UpgC
DFR85_RS28550 ABC sugar transporter ATPase MalK
DFR85_RS28565 Extracellular solute−binding protein TreS
DFR85_RS28570 ABC sugar transporter permease TreT
DFR85_RS28575 ABC sugar transporter permease TreU
DFR85_RS28580 ABC sugar transporter ATPase TreV
DFR85_RS13445 MFS sugar/acid transporter MSAT1
DFR85_RS21375 MFS sugar/acid transporter MSAT2
DFR85_RS28395 MFS sugar/acid transporter MSAT3
DFR85_RS28400 MFS sugar/acid transporter MSAT4
DFR85_RS28490 MFS sugar/acid transporter MSAT5
DFR85_RS29280 MFS sugar/acid transporter MSAT6
DFR85_RS30810 MFS sugar/acid transporter MSAT7
DFR85_RS30900 MFS sugar/acid transporter MSAT8
DFR85_RS30905 MFS sugar/acid transporter MSAT9
DFR85_RS30910 MFS sugar/acid transporter MSAT10
DFR85_RS30985 MFS sugar/acid transporter MSAT11
DFR85_RS31345 MFS sugar/acid transporter MSAT12
DFR85_RS31490 MFS sugar/acid transporter MSAT13
DFR85_RS21590 MFS sugar/acid transporter MSAT14
DFR85_RS16765 Extracellular solute−binding protein ESB1
DFR85_RS18800 Extracellular solute−binding protein ESB2
DFR85_RS27095 Extracellular solute−binding protein ESB3
EX
ST
AN
AE
EX vs ST
AN vs EX
AN vs ST
AE vs EX
AE vs ST
AE vs AN
Normalized protein abundance
0
3□10^10
6□10^10
9□10^10
Log2 fold change
−10
−5
0
5
10

Supplementary References

1. Laska, S., Lottspeich, F. & Kletzin, A. Membrane-bound hydrogenase and sulfur reductase of the hyperthermophilic and acidophilic archaeon *Acidianus ambivalens*. *Microbiology* **149**, 2357–2371 (2003).
2. Bertero, M. G. *et al.* Insights into the respiratory electron transfer pathway from the structure of nitrate reductase A. *Nature Structural Biology* **10**, 681–687 (2003).
3. Volbeda, A. *et al.* Crystal structure of the O_2-tolerant membrane-bound hydrogenase 1 from *Escherichia coli* in complex with its cognate cytochrome *b*. *Structure* **21**, 184–190 (2013).
4. Bernhard, M., Benelli, B., Hochkoeppler, A., Zannoni, D. & Friedrich, B. Functional and structural role of the cytochrome *b* subunit of the membrane-bound hydrogenase complex of *Alcaligenes eutrophus* H16. *European Journal of Biochemistry* **248**, 179–186 (1997).
5. Watanabe, T. *et al.* Three-megadalton complex of methanogenic electron-bifurcating and CO_2-fixing enzymes. *Science* **373**, 1151–1156 (2021).
6. Grinter, R. *et al.* Structural basis for bacterial energy extraction from atmospheric hydrogen. *Nature* **615**, 541–547 (2023).
7. Yankovskaya, V. *et al.* Architecture of succinate dehydrogenase and reactive oxygen species generation. *Science* **299**, 700–704 (2003).
8. Nauer, P. A., Chiri, E., Jirapanjawat, T., Greening, C. & Cook, P. L. M. Technical note: inexpensive modification of exetainers for the reliable storage of trace-level hydrogen and carbon monoxide gas samples. *Biogeosciences* **18**, 729–737 (2021).
9. Novelli, P. C. C. *et al.* Molecular hydrogen in the troposphere: global distribution and budget. *Journal of Geophysical Research Atmospheres* **104**, 30427–30444 (1999).
10. Cordero, P. R. F. *et al.* Atmospheric carbon monoxide oxidation is a widespread mechanism supporting microbial survival. *ISME Journal* **13**, 2868–2881 (2019).
