# Supplementary material for: Trace gas oxidation sustains energy needs of a thermophilic archaeon at suboptimal temperatures
Source: Nat Commun. 2024 Apr 15;15:3219. doi: 10.1038/s41467-024-47324-2 (PMC11018855; doi:10.1038/s41467-024-47324-2)
Supplement: Supplementary file 3 — Description of Additional Supplementary Files [file 41467_2024_47324_MOESM3_ESM.pdf]

Description of Additional Supplementary Files:

Supplementary Dataset 1: (xlsx). Gas chromatography measurements of H2, CO, and CH4 concentrations in tested cultures and negative controls.

Supplementary Dataset 2: (xlsx). Measurements of survival assay.

Supplementary Dataset 3: (xlsx). Group 1 and 2 [NiFe] hydrogenases used for phylogenetic analysis.

Supplementary Dataset 4: (xlsx). Annotations and protein expressions of predicted genes of Acidianus brierleyi.

Supplementary Dataset 5: (zip). Full maximum-likelihood phylogenetic tree of amino acid sequences of uptake group 1 and 2 [NiFe]-hydrogenases large/catalytic subunit identified in A. brierleyi (4 sequences; red text), genomes of all archaeal representative species in Genome Taxonomy Database (GTDB) release 202 (202 sequences), and hydrogenase reference database HydDB (1003 sequences). Group 3 and 4 [NiFe]-hydrogenases were included as outgroups and the phylogeny was rooted between group 4 [NiFe]-hydrogenases and all other groups. Subgroups/clades that were exclusively bacterial or archaeal are shaded in grey or pink, respectively. Details on alignment and tree inference can be found in Methods and all sequences are provided in Supplementary Dataset 3. Each node was colored by ultrafast bootstrap support percentage (1000 replicates) and the scale bar indicates the average number of substitutions per site.

Supplementary Dataset 6: (Zip). AlphaFold2 models of the four Acidianus hydrogenases.
