# Supplementary figures and images for: Trace gas oxidation sustains energy needs of a thermophilic archaeon at suboptimal temperatures
Source: Nat Commun. 2024 Apr 15;15:3219. doi: 10.1038/s41467-024-47324-2 (PMC11018855; doi:10.1038/s41467-024-47324-2)

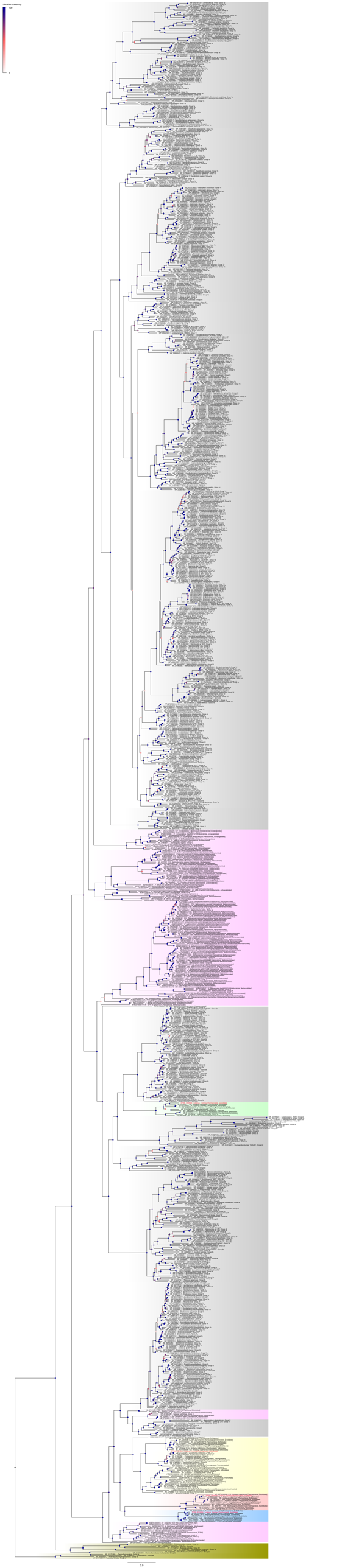

Supplement: Supplementary file 8 — Supplementary Dataset 5 [file 41467_2024_47324_MOESM8_ESM.zip › Figure S4. Full hydrogenase tree.pdf]
