# Supplementary material for: Trace gas oxidation sustains energy needs of a thermophilic archaeon at suboptimal temperatures
Source: Nat Commun. 2024 Apr 15;15:3219. doi: 10.1038/s41467-024-47324-2 (PMC11018855; doi:10.1038/s41467-024-47324-2)
Supplement: Supplementary file 10 — Reporting Summary [file 41467_2024_47324_MOESM10_ESM.pdf]

nature portfolio

Corresponding author(s): Chris Greening & Pok Man Leung

Last updated by author(s): Feb 26, 2024

Reporting Summary

Nature Portfolio wishes to improve the reproducibility of the work that we publish. This form provides structure for consistency and transparency in reporting. For further information on Nature Portfolio policies, see our Editorial Policies and the Editorial Policy Checklist.

Statistics

For all statistical analyses, confirm that the following items are present in the figure legend, table legend, main text, or Methods section.

n/a	Confirmed	
☐	☒	The exact sample size (*n*) for each experimental group/condition, given as a discrete number and unit of measurement
☐	☒	A statement on whether measurements were taken from distinct samples or whether the same sample was measured repeatedly
☐	☒	The statistical test(s) used AND whether they are one- or two-sided<br>*Only common tests should be described solely by name; describe more complex techniques in the Methods section.*
☒	☐	A description of all covariates tested
☐	☒	A description of any assumptions or corrections, such as tests of normality and adjustment for multiple comparisons
☐	☒	A full description of the statistical parameters including central tendency (e.g. means) or other basic estimates (e.g. regression coefficient) AND variation (e.g. standard deviation) or associated estimates of uncertainty (e.g. confidence intervals)
☐	☒	For null hypothesis testing, the test statistic (e.g. *F*, *t*, *r*) with confidence intervals, effect sizes, degrees of freedom and *P* value noted<br>*Give P values as exact values whenever suitable.*
☒	☐	For Bayesian analysis, information on the choice of priors and Markov chain Monte Carlo settings
☒	☐	For hierarchical and complex designs, identification of the appropriate level for tests and full reporting of outcomes
☒	☐	Estimates of effect sizes (e.g. Cohen's *d*, Pearson's *r*), indicating how they were calculated

Our web collection on statistics for biologists contains articles on many of the points above.

Software and code

Policy information about availability of computer code

Data collection	- VICI Trace Gas Analyser (TGA) integration software (Valco Instruments Co. Inc., USA)<br>- QExactive HF mass spectrometer (ThermoFisher Scientific)<br>- BioSpectrometer Basic (Eppendorf)
Data analysis	No custom algorithm/software was used for the data analysis.<br><br>Databases used for analyzing sequence data:<br>- NCBI Conserved Domain Database (CDD) v3.19<br>- Pfam protein family database v34.0<br>- KEGG protein database (accessed 22 November 2021)<br>- HydDB (https://services.birc.au.dk/hyddb/)<br>- Genome Taxonomy Database (GTDB) release 202<br>- PDB database<br><br>Data were analysed using the following open-sourced or commercial software:<br>- BLAST+ v2.9.0<br>- PfamScan v1.6<br>- PSORTb v3.0.3<br>- DRAM v1.2.4<br>- gggenes v0.4.1<br>- Clustal Omega v1.2.4

- HMMER v3.3.2
- MAFFT-L-INS-i v7.505
- IQ-TREE2 v2.2.0
- AlphaFold multimer v2.1.1
- GraphPad Prism v9.3.1
- Percolator
- Fragpipe software suite v17.1
- MSFragger search engine v3.4
- LFQ-Analyst
- limma

For manuscripts utilizing custom algorithms or software that are central to the research but not yet described in published literature, software must be made available to editors and reviewers. We strongly encourage code deposition in a community repository (e.g. GitHub). See the Nature Portfolio guidelines for submitting code & software for further information.

Data

Policy information about availability of data

All manuscripts must include a data availability statement. This statement should provide the following information, where applicable:

- Accession codes, unique identifiers, or web links for publicly available datasets
- A description of any restrictions on data availability
- For clinical datasets or third party data, please ensure that the statement adheres to our policy

The mass spectrometry proteomics data have been deposited to the ProteomeXchange Consortium via the PRIDE partner repository with the dataset identifier PXD040286. All other study data are included in the article and/or supporting information. Source data are provided with this paper.

Research involving human participants, their data, or biological material

Policy information about studies with human participants or human data. See also policy information about sex, gender (identity/presentation), and sexual orientation and race, ethnicity and racism.

Reporting on sex and gender	There is no involvement of human research participants in this study
Reporting on race, ethnicity, or other socially relevant groupings	There is no involvement of human research participants in this study
Population characteristics	There is no involvement of human research participants in this study
Recruitment	There is no involvement of human research participants in this study
Ethics oversight	There is no involvement of human research participants in this study

Note that full information on the approval of the study protocol must also be provided in the manuscript.

Field-specific reporting

Please select the one below that is the best fit for your research. If you are not sure, read the appropriate sections before making your selection.

☒ Life sciences ☐ Behavioural & social sciences ☐ Ecological, evolutionary & environmental sciences

For a reference copy of the document with all sections, see nature.com/documents/nr-reporting-summary-flat.pdf

Life sciences study design

All studies must disclose on these points even when the disclosure is negative.

Sample size	No sample size calculation was performed. Sample sizes were chosen to support meaningful conclusions with the consideration of cost-efficiency.
Data exclusions	No data were excluded from the analysis.
Replication	For hydrogen and carbon monoxide consumption measurement, cultures were prepared in triplicate. For hydrogen consumption kinetics experiment and whole cell shotgun proteomics, quadruplicate cultures were used. The reproducibility of the experiment was reflected by the consistency of the replicates used. Activity staining of hydrogenases was replicated with independent experiments twice with same results.
Randomization	Randomization is not applicable to this study as the cultures and replicates were prepared and analysed identically.
Blinding	Blinding is not applicable to this study as the cultures and replicates were prepared and analysed identically.

Reporting for specific materials, systems and methods

We require information from authors about some types of materials, experimental systems and methods used in many studies. Here, indicate whether each material, system or method listed is relevant to your study. If you are not sure if a list item applies to your research, read the appropriate section before selecting a response.

Materials & experimental systems

n/a	Involved in the study
☒	☐ Antibodies
☒	☐ Eukaryotic cell lines
☒	☐ Palaeontology and archaeology
☒	☐ Animals and other organisms
☒	☐ Clinical data
☒	☐ Dual use research of concern
☒	☐ Plants

Methods

n/a	Involved in the study
☒	☐ ChIP-seq
☒	☐ Flow cytometry
☒	☐ MRI-based neuroimaging

Plants

Seed stocks	There is no involvement of plants in this study
Novel plant genotypes	There is no involvement of plants in this study
Authentication	There is no involvement of plants in this study
